# The Economic Costs of Childhood Disability: A Literature Review

**DOI:** 10.3390/ijerph18073531

**Published:** 2021-03-29

**Authors:** Ahmed Ramadan Shokry Shahat, Giulia Greco

**Affiliations:** 1 Quality and Accreditation Directorate, Ministry of Health, Sulaibikhat 13001, Kuwait; 2 Department of Global Health and Development, London School of Hygiene and Tropical Medicine, Keppel Street, London WC1E 7HT, UK; giulia.greco@lshtm.ac.uk

**Keywords:** child, disability, burden, cost

## Abstract

Background: This literature review investigates the economic costs of childhood disability analysing methodologies used and summarizing the burden worldwide comparing developed and developing countries. Methods: Four electronic databases were searched. Studies were categorised according to country, perspective, methods of costing, disability category, and time horizon. Annual costs were converted to 2019 current US dollars then compared to the country’s per capita current health expenditure (CHE) and gross domestic product (GDP). Results: Of 2468 references identified, 20 were included in the review. Annual burden of childhood disability ranged ≈$450–69,500 worldwide. Childhood disability imposes a heavy economic burden on families, health systems, and societies. The reason for the wide range of costs is the variability in perspective, costs included, methods, and disability type. Conclusion: The annual societal costs for one disabled child could be up to the country’s GDP per capita. The burden is heavier on households in developing countries as most of the costs are paid out-of-pocket leading to impoverishment of the whole family. Efforts should be directed to avoid preventable childhood disabilities and to support disabled children and their households to make them more independent and increase their productivity. More studies from developing countries are needed.

## 1. Introduction

Around one billion (15% of the world population) have some level of disability according to the World Health Organization (WHO) and World Bank [1,2]. There is currently no reliable estimate for the number of disabled children around the world [3,4]; however, estimates vary between 0.4–12.7% [5]. This is partly due to differences in defining disability. The more accepted approach in defining disability is the “bio-psycho-social model” which defines disability not only based on the individual’s health condition, but rather the interaction between the health condition and contextual factors (environmental/personal) [1].

Research shows an association between poverty and disability [6,7,8,9] but the relationship is complex and moves in both directions. A disabled child has less access to schooling and lower probability of continuing to higher education which means less human capital accumulation leading to unemployment or lower paid jobs and inevitably leading to chronic poverty [1,10,11,12]. Childhood disability can also impoverish the household because of direct extra costs of caring for the disabled child (e.g., healthcare, wheelchair, child care) and indirect costs (e.g., job loss to care for the child or having to work part-time/ flexible hours) [6,13,14,15,16,17]. On the other hand, poverty is a risk factor for childhood disability. Poor nutrition and water sanitation, unsafe antenatal care, and high prevalence of preventable diseases and accidents among less advantaged individuals increases the risk of childhood and adult disability especially in developing countries [3,13]. There is also a big disparity between evidence arising from developed versus developing countries regarding the association between poverty and disability [1,3,6,10].

Cost of illness studies have been published since the late 1950s. Reference [18] in 1967, Dorothy Rice, proposed a model for cost of illness that differentiates direct and indirect costs [19]. The choice of what costs to include, in the present and in the future, is subject to debate [20]. The perspective of the costing study (patient/household, health system, or society) plays an important role in what costs to include or exclude from the analysis. Recent efforts have been made to provide guidelines for healthcare costing studies. The most notable is the “Reference Case for Estimating the Costs of Global Health Services and Interventions” by the Global Health Cost Consortium (GHCC) launched in 2016. The reference case aims at improving consistency and transparency of methods, assumptions, and reporting [21].

Previous reviews discussed the costs of disability in children [22,23,24] but none could calculate figures due to lack of consistency in methods for defining disability and estimation of costs. To the best of our knowledge, no reviews have compared developed vs. developing countries.

This research intends to be the first to study the costs of childhood disabilities comparing country, perspective, methods of costing, disability category, and time horizon. Beside reporting costs, we analyse the methodologies used in different studies for calculating cost of illness and their relevance to the setting and perspective. We also compared annual costs to the current health expenditure per capita and to the GDP per capita to show the magnitude of the burden.

The aim of this work is to investigate the costs of childhood disability in developed and developing countries through a literature review. The objectives are:

1. To identify costs of childhood disability from selected references and categorize them according to setting, type of disability, perspective, and time horizon.
2. To quantify the burden of disability on the health system and society by comparing the cost to current health expenditure (CHE) per capita and gross domestic product (GDP) per capita.

To provide critical qualitative comparison between developed and developing countries.

## 2. Materials and Methods 

A literature review was conducted largely following Preferred Reporting Items for Systematic Reviews and Meta-Analysis (PRISMA) guidelines [25,26]. Only one researcher performed the initial search, selection of articles, and critical appraisal (AS). All previous steps were then revised, corrected, and approved by the second researcher (GG). However, the two researchers did not perform the evaluation independently hence it is considered a literature review not a systematic review. 

Literature was searched for the key terms of cost, child, and disability. Alternative search terms were also used for each key word. Selecting the search terms for disability was challenging as there is a wide range of medical conditions which may cause disability. In this review, we used keywords general to the concept of disability. In addition, we searched for three of the most common medical conditions causing childhood disability; cerebral palsy (CP), autism spectrum disorders (ASD), and Down syndrome (DS). These medical conditions were selected because they almost always occur/manifest in childhood, cause considerable disability, and have long survival so the costs can be followed for a longer time.

Inclusion criteria were: peer reviewed journal articles, from 1980 onwards, in the English language only, estimating any type of economic costs of children (up to the age of 18), with any type of disability.

Countries were categorised as developed or developing. All high-income countries were considered “developed”, all low-and-middle-income countries (LMIC) were considered “developing”. Ranking of countries’ income levels was taken from the latest World Bank country and lending group ranking [27].

### 2.1. Searching Strategy and Databases

The databases Medline, Embase, EconLit, and Global Health were searched. Alternative search strategies were also used through snowball sampling [28] where the search strategy evolves based on the relevant literature identified through the initial electronic search. This included searching reference lists, hand searching relevant journals and author searching. Details of the search strategies on the four databases is available in Appendix A,Appendix B,Appendix C,Appendix D.

### 2.2. Identifying Relevant Literature

The searching and sifting process was performed largely following PRISMA guidelines [26]. The results of electronic database searches were uploaded and managed using Mendeley reference management software and duplicates were removed. Titles were screened against inclusion criteria. After exclusion of irrelevant titles, abstracts of the remaining references were screened again. The full text of the remaining references was retrieved, and critical appraisal of their quality was performed. At this stage, more full text articles were included through snowball sampling while some studies were excluded because full text could not be found.

### 2.3. Data Extraction

A data extraction form was designed using google-docs. The form had two sections, one for data extraction and another for the critical appraisal questions. Data were then exported into an excel file for analysis. The form can be viewed at https://forms.gle/ntoEivcgT5xN7Agd9 (accessed on 13 July 2019).

### 2.4. Categorizing Costs

Types of costs were categorised into the following:

- Direct-medical: costs for diagnosis, treatment, managing complications of the condition causing the disability.
- Direct non-medical: includes special education, rehabilitation, and transportation costs.
- Indirect non-medical: productivity loss and time lost by parents due to caring for the disabled child.
- Future related: medical costs related to the condition causing disability that happen in the future years.
- Future unrelated: includes future productivity loss by the disabled children and their caregivers.

### 2.5. Critical Appraisal

A modification of the 10-point checklist by Drummond et al. [29] was used to evaluate the quality of the articles. The checklist was originally designed to critically appraise economic evaluation studies, hence rephrasing and excluding some questions was necessary. The modified checklist contained 20 yes/no questions. All questions were given equal weight. Studies scoring yes in more than 75% of the applicable questions were considered to have a low risk of bias, whereas, those scoring (50–75%) a moderate risk of bias, and studies scoring less than 50% a high risk of bias. 

When an item is partially met it was considered (yes). However, all items were reviewed again by the second researcher (GG) and discussed to reach a final decision on assigning the question a yes/no answer. The critical appraisal tool is available in Appendix E.

### 2.6. Synthesis of Evidence

Qualitative comparison was made between studies by categorising and analysing them according to the following items:

○ Country (name, developed/developing)
○ Perspective (household, health system, or societal perspective)
○ Methods of costing
○ Disability category (types of disability/medical condition)
○ Time horizon

The data extraction table is available in Appendix F.

For quantitative comparison, annual costs reported in the studies were compared to the CHE per capita of the country obtained from the latest estimates published by the WHO in 2016 [30] and then the figures were inflated to 2019 US$. Comparison was also made with GDP per capita for the country obtained from the International Monetary Fund (IMF) projections for GDP published in the World Economic Outlook Database in April 2019 [31]. Lifetime costs were transformed to annual costs by dividing the lifetime costs by the number of years reported in the study model. The ratios of the annual costs to CHE and GDP were displayed as percentages in the results table. The ratios were displayed in a scatterplot after they were transformed to natural logs to minimize the scale and make it easier for display. This means that the midline (zero) corresponds to 100%, i.e., the estimated cost is equal to the CHE/GDP per capita. All the data point to the right of the midline (positive) are multiples of the CHE/GDP per capita i.e., the costs are more than CHE/GDP per capita. Any data points to the left of the midline (negative) are fractions of the CHE/GDP per capita i.e., the costs are less than the CHE/GDP per capita. The studies were arranged chronologically from oldest (bottom) to newest (top) to show if there was a trend over time. A different colour was given to data points for each of the three perspectives (the household, the health system, and the societal). A different shape was given to data points to differentiate annual costs reported in the original studies from annual costs that we estimated from the lifetime costs. As some studies estimated separate costs for more than one type of disability, each data point on the scatter plot represents a cost estimate rather than a study.

### 2.7. Summarizing Results and Currency Conversion

For comparability, all results were displayed in 2019 US dollars (US$). Costs from the original studies were first inflated to 2019 local currency values using local currency inflation rate from consumer price index of the international monetary fund [32]. Costs in currencies other than US$ were then converted to 2019 US$ using the exchange rate of the base year (July 2019 conversion rate).

Costs were reported as average (mean) cost whenever possible. If both mean and median are reported; the mean was chosen. Because the 95% confidence interval was not available in all studies, it was not reported in our results. Costs were rounded and presented as approximate figures (≈) to emphasize the idea that these costs need to be compared categorically rather than statistically. However, the actual (non-rounded) estimates are presented in the results table.

## 3. Results

### 3.1. Finding Relevant Studies

**Figure 1:**
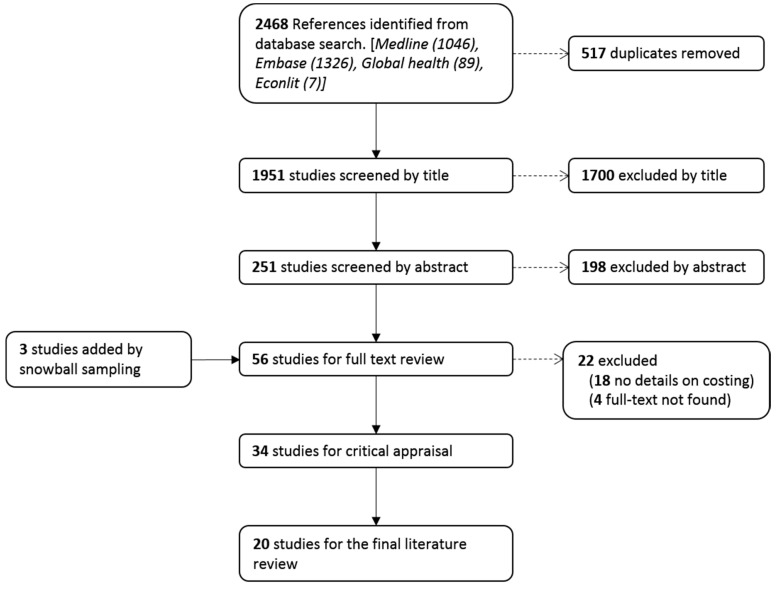
Literature selection process.

The initial literature search identified 2468 references. After removal of 517 duplicates, the titles of the remaining 1951 references were screened. Of these, 251 were retained for abstract review applying the inclusion and exclusion criteria. After revising the abstracts, 53 references were selected for full text review and a further three were found through snowball sampling. The critical appraisal tool was applied to 34 studies, 17 were found to have low risk of bias and were, therefore, included in this review. Only two studies from this group were from a developing country. Fourteen studies had moderate risk of bias and were, therefore, excluded. We exceptionally included three studies from the moderate risk of bias group as they were from developing countries, so they were needed for the sake of comparison. All studies with high risk of bias were excluded (*n* = 3). This resulted in 20 studies that were included in the final analysis. Details of the sifting process are shown in Figure 1.

### 3.2. Summary of the Results

The annual costs of childhood disability reported in the studies ranged ≈$450–69,500. In developing countries, the costs ranged ≈$500–7500 while in developed countries it ranged ≈$450–69,500.

The lifetime costs ranged ≈$41,000–4,300,00 worldwide. In developing countries, costs ranged ≈$41,000–91,000 while in developed countries the range was ≈$32,000–4,300,000.

**Figure 2:**
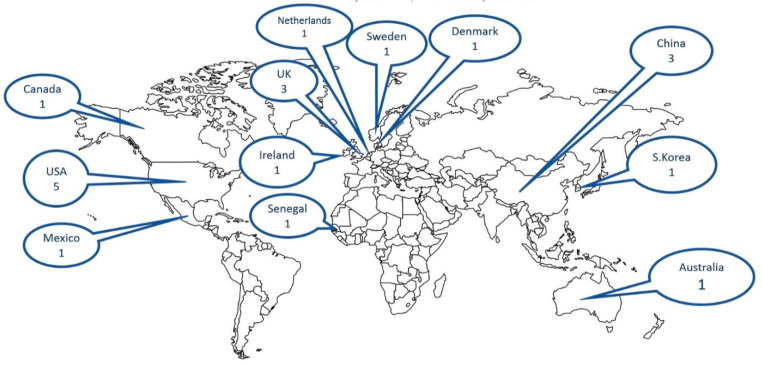
Geographical distribution of the review studies This is a royalty free image, ©Copyright J. Bruce Jones 2019 www.mapsfordesign (accessed on 26 August 2019).

The 20 studies were spread across 12 countries in five continents. The majority (n = 15) were set in developed countries. Figure 2 shows the distribution of the reviewed studies over the world map.

Regarding type of disability; six studies estimated the costs of disability in general or reported the costs of more than one category of disability. Six studies focused on the costs of autism spectrum disorders (ASD), four on cerebral palsy (CP), and three on Down syndrome (DS). Only one study estimated the costs of disability as a sequel of meningitis.

Regarding study perspective; the societal perspective was considered in 10 studies, the household perspective in six, while four studies were from the health system perspective. 

Regarding time horizon; more than half of the studies (*n* = 12) reported the annual costs, whereas six estimated the lifetime costs. The study by Solmi et al. [33] was the only one to report weekly extra costs. Barrett et al. [34] reported costs over a six-month period. Kageleiry et al. [35] presented the aggregate costs for the whole childhood period (0–18 years) which was difficult to break down into annual costs but could, nevertheless, be compared to lifetime costs regarding the methodology.

A summary of the findings from the 21 studies is presented in Table 1.

#### General Methodological Findings

The definition of disability varied between the reviewed studies. The majority of the studies (*n* = 14) identified disabled children based on a confirmed diagnosis of a medical condition, whereas only three studies used a definition that follows the bio-psycho-social model. Three studies were not clear on the method of defining disability.

The perspective of the analysis was determinant in the choice of study methods and the types of cost included. The societal perspective was more commonly used in developed countries. Nine out of 15 of the studies from developed countries focused on the societal perspective, five were from the health system perspective and only one was from the household perspective. In fact, seven out of eight studies from European Union (EU) countries were from a societal perspective. In developing countries, four out of five studies were from the household perspective, only one from the societal perspective and none from the health system perspective.

The most frequently measured costs were direct medical and direct non-medical costs which were estimated in 17 and 16 studies, respectively. Indirect non-medical costs were estimated in 11 studies, future-related (medical) costs in six, and future unrelated costs in only five studies.

Whenever the method of estimating indirect non-medical costs is explicitly mentioned it was the human capital approach. The only exception in developed countries was the novel use of compensating variation method by Solmi et al. [33] The friction cost method was not used in any of the reviewed studies and was not found in any of the 34 studies included in the critical appraisal. Two studies from developing countries estimated productivity loss by directly asking parents on their estimation of income forgone rather than the standard estimation through the human capital approach method [36,37].

### 3.3. Lifetime Cost Studies

The lifetime costs ranged ≈$41,000–4,300,00 this corresponds to annual costs of ≈$450–69,500. Six studies estimated lifetime costs of disability. These studies were included even though cost estimation continued into adulthood because it is a long-term consequence of disability acquired at birth or during childhood. Four of the studies were from the societal perspective, one from the health system perspective, and one from the household perspective.

The method of estimation of lifetime costs was similar in the six studies; the incidence/prevalence of the condition is estimated, then average costs at each age band till the end of life expectancy is estimated. This estimate is then used to model the lifetime costs for a hypothetical person (or cohort) who acquires the condition at the base year and dies at the end of life expectancy. Therefore, we considered lifetime costs studies to be model-based studies to differentiate them from other studies which estimate annual costs in real-time in the current year(s) i.e., survey-based.

Discounting was used for future costs (ranging 3–5%) in all the lifetime studies. Sensitivity analysis to account for uncertainty around the measures estimated in the study was used in five of the six studies.

The study by Griffiths et al. [36] on lifetime costs of meningitis sequelae in children in Senegal was the only one from a developing country and was the only study to be truncated at the age of 30 years, while the remaining five studies were from developed countries and truncated at an age not far from the average life expectancy of the population.

The six studies focused on a single disease or a group of very similar disorders; three on CP, two on ASD, and one on meningitis sequelae. Regarding data sources, two studies pooled data from different studies to estimate the average costs, [38,39] two used national register data, [40,41] and two estimated costs based on parents’ reported expenditures and healthcare utilization [36,42].

### 3.4. Magnitude of the Burden

It was not possible to compare the studies quantitatively because of methodological differences. However, we attempted to estimate the ratios of annual costs to their country’s own per capita CHE and GPD and display it graphically in a scatter plot. This plot would allow the reader to visualise results in a comparable way and show the general differences between countries and between perspectives. On the scatterplot, annual costs estimated through survey-based methods were given a different shape (triangle) to differentiate them from annual costs that we estimated from the reported lifetime costs studies model-based method (circle).

**Figure 3:**
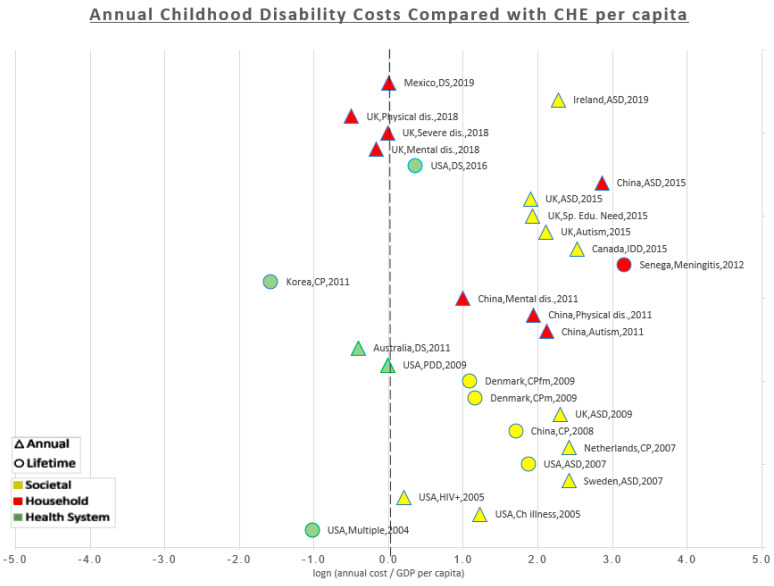
Annual childhood disability costs compared with CHE per capita. Care must be taken not to simply compare the costs from different studies as there are considerable methodological differences between them, this graph is just a way of summarizing these differences.

Figure 3 is a scatterplot that shows the ratio of each estimated cost to the country’s CHE per capita. All the costs estimated from the societal perspective (yellow) were to the right of the midline, i.e., higher than CHE per capita. From the household perspective (red), costs estimated in China and Senegal were higher than CHE per capita, costs from Mexico were equal to CHE per capita, and costs from the UK were equal or less than CHE per capita. From the health system perspective (green), costs were generally lower compared to the other perspectives. Costs estimated in Korea and Australia were lower than CHE per capita. Three cost estimates from the USA in the years 2004, 2011, and 2016 were lower than, equal, and higher than the CHE per capita respectively. This may indicate a trend of rising healthcare costs in the USA; however, type of disability and methods could be major confounding factors.

**Figure 4:**
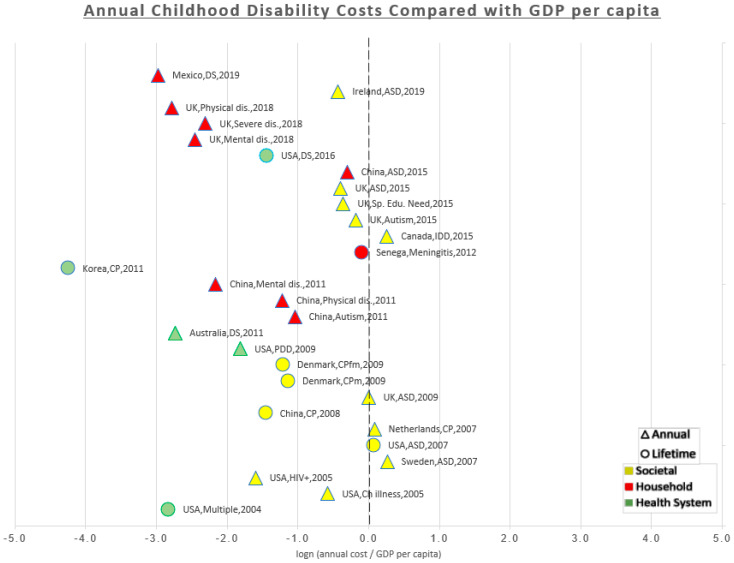
Annual childhood disability costs compared with GDP per capita. Care must be taken not to simply compare the costs from different studies as there are considerable methodological differences between them, this graph is just a way of summarizing these differences.

Figure 4 is a scatterplot that shows the ratio of each estimated cost to the country’s GDP per capita. All costs estimated from the health system (green) and the household perspective (red) were lower than their country’s GDP per capita. From the societal perspective (yellow), costs estimated in the UK, the Netherlands, Canada, Sweden, and one USA study were equal or more than GDP per capita, while the other costs estimated from the societal perspective in Ireland, USA, China, Denmark, and some UK studies were lower than one GDP per capita.

## 4. Discussion

All reviewed studies concluded that there is a substantial economic burden attributed to childhood disability regardless of the study perspective or setting. The household of the disabled child bears a heavy economic burden. This is either in the form of out-of-pocket (OOP) expenditures, or opportunity costs due to productivity time lost caring for the disabled child. This is true even in countries with public coverage of health, education, and social services. This economic burden is shared by the family of the disabled child, public health services, and the society. The share of each party is variable depending on the health and welfare system in the study setting. In developing countries, much of the burden is on the household in the form of OOP expenditures and lost productivity. In Mexico, Martınez-Valverde et al. found that 33% of families with DS children had catastrophic expenses and 46% of the families had to borrow money to pay for medical expenses [48].

In developed countries, part of the burden is shifted to the public services (e.g., health, special education, and disability benefits) but there is still a substantial burden on the families. In the UK, an additional annual amount of ≈$4200 is needed for the families of a severely disabled child to have the same living standard of their matched families without a disabled child [33,53].

### 4.1. Definition of Disability

The WHO report on disability recommended the bio-psycho-social model for defining disability as an interaction between the physical condition and the environment [1]. This was only used in three of the 20 reviewed studies while the majority used confirmed clinical diagnosis of a certain disease as the indication of disability. However, this may be biased because the literature search was done in databases which focus primarily on medical rather than social research.

The definition of disability varies globally, even within some countries there is no consensus on the national definition which explains why different studies produce different prevalence rates for the same population. In the USA, Newacheck et al. demonstrated that the prevalence of childhood disability may range from 7.3% to 30% depending on the definition of disability used [49]. Thus, it is important for researchers to use uniform definitions for disability to allow results to be comparable and be more relevant and informative for planning and budgeting of services.

### 4.2. Human Capital vs. Friction Cost

Valuing indirect costs, especially childcare time and lost productivity, has been an issue of much debate. As they are less straightforward to measure and more complicated to explain for decision makers, researchers have used different methods to estimate these costs. Among 11 studies that considered indirect non-medical costs, the human capital approach was the most common method. Although theoretically it may be superior to the human capital approach, the friction cost method was never used in any of the reviewed studies. This may be due to the limited use of the friction cost method in healthcare literature and that its costs are not readily available [20]. We suggest also that the human capital approach could be easier to present and explain to decision makers and the public.

Three studies used alternative methods. A British study used propensity score matching and compensating variation method which calculates ”*the amount of additional income a family with a disabled child would require to achieve the same living standards as a similar family without a disabled child*” [53]. In a Senegalese study on effects of meningococcal meningitis, the opportunity cost of time lost by parents’ due to their child’s illness was reported and valued by the parents [36]. This approach was suggested by the researchers to be more suitable to LMIC settings. A similar approach was used in China, where parents replied to open questions such as “*How much total income do you expect that all family members would have earned in the past year if your child did not have the disease?*” [37].

The use of compensating variation is promising and could provide good evidence for evaluating the effectiveness of disability benefits in the country as it estimates the amount needed to fill the gap in standard of living between families with/without a disabled child. However, it can not be used to estimate societal cost as the focus here is the household.

We would still recommend the use of human capital approach as it is the most commonly used method and would make results comparable to other studies.

### 4.3. Societal vs. Household vs. Health System Perspective

The perspective may be the most determinant factor in a costing study as it would dictate what costs to include/exclude, where to collect the data from, and who is the audience of the study. No perspective can be claimed to be superior to others, it depends on the context. Careful analysis of the health system of the country of the study and the target audience should lead to the selection of the most suitable perspective.

In EU countries, disabled children can receive good quality of public healthcare, special education, and social services at no or very minimal costs. This explains why most studies in EU countries focus on the social perspective as most of the disability burden falls on public services. In this context, the household perspective would underestimate the negative effects of disability because a considerable part of the burden is shifted from the family to the social welfare network.

The healthcare system in the USA differs from that in Europe as there is no single national or social health insurance in the USA. Disabled children can receive healthcare through Medicaid, private insurance, or could even have no healthcare coverage at all. Because of the variable and more complex health system in the USA, different studies adopt different perspectives to cover all viewpoints. Three out of five American studies focused on the health system perspective. This reflects the growing concern by the healthcare payers (public or private) about the rising healthcare costs in the USA.

In developing countries, public services are of lower quality and limited coverage leading to higher reliance on private providers. This leads to significant OOP costs for the families. This explains why four out of five studies from developing countries focused on the household perspective while it was used in only one out of 15 studies in developed countries. Limited expertise in costing in developing countries, poor documentation, and unavailability of sources of cost data may also be factors deterring researchers in those countries from taking a wider perspective.

It was very difficult to aggregate the methods used into meaningful categories for several reasons. The methods used to calculate each cost category were variable, but what made it even more challenging is that many studies lacked the level of detail needed for such analysis. We have included in Appendix F some more details from the studies on their methods.

### 4.4. Developed vs. Developing

Studies originating from developing countries were fewer in number and were generally of lower quality compared to studies from developed countries. Out of 34 studies included in full-text critical appraisal, eight were from developing countries, and only two had low risk of bias. Studies from developing countries focused more on the household and OOP expenses. The health system perspective was never used in studies from developing countries. This may indicate lack of interest from decision makers in this kind of studies and may also indicate the difficulty in collection of health system costs in these sittings.

Methods in studies from developing countries were less explicit especially in describing sources of each cost component and how it was calculated. Costs were generally lower in developing countries, annual costs ranged ≈$500–7500 while in developed countries they were ≈$2600–69,000. This may be partly because the cost of healthcare is lower in these countries and partly due to methodological differences (e.g., fewer costs included).

The estimation of lifetime societal costs of CP in two studies, one in China (≈$91,000) [42] and another in Denmark (≈$1.2–1.3 million) [40], shows a huge difference. Although both studies used the same perspective, the Danish study relied on national register data (including all patients registered since the 1960s) and was very explicit on details of costs included, sources of costs, and how they were calculated. The Chinese study was based on interviews with 319 parents of CP children to ask about utilization and expenditure related to their child disability as well as productivity loss. Less details were offered in the Chinese study on how each cost component was calculated. This may partly explain why both studies came to very different numbers that cannot simply be attributed to differences in healthcare costs or wage rates.

This shows that the choice of the most suitable method may vary between countries as it should account for differences in health systems (what services are covered by insurance or public services and what services are paid by the families), and should also account for the availability of data and their quality. The lack of availability of reliable population-level cost data in developing countries makes the results of the studies less accurate and less generalizable. This highlights the need for guidelines that set standards for conducting and reporting cost studies which allows them to be compared across time and countries. The guideline should be comprehensive yet flexible to be adapted to context and setting. A notable effort in this field was undertaken by the Global Health Cost Consortium (GHCC) in the Reference Case for Estimating the Costs of Global Health Services and Interventions that *“adopts a “comply or justify” approach, which allows the analyst to adapt to the specific requirements of the costing exercise, but introduces the condition that judgments about methods choices are made explicitly and transparently”* [21].

### 4.5. Mean vs. Median

Cost data in healthcare are typically positively skewed (right tailed). This is because a few patients (very severe or complicated cases) use significantly more resources than the rest which increases the mean cost. Therefore, the median is statistically more appropriate to describe cost data and non-parametric techniques are more appropriate for analysis. Nevertheless, decision makers are interested in average (mean) cost not the median [29] because average cost is what they would use in budgeting. Different statistical techniques have been used for presenting the cost results in mean costs including bootstrapping and using the gamma distribution [29,54].

Most reviewed studies reported the mean cost. One study reported both mean and median cost, [45] allowing readers interested in the expected cost for an individual patient to know the median while presenting the mean for decision makers who are more interested in average cost for the population. In this review, we assumed that mean costs are more relevant in describing the burden on the health system and society. Thus, the mean costs were reported in this review whenever available.

We recommend that the ideal practice would be reporting both mean and median costs. If the mean is used, care should be taken to use the appropriate statistical techniques to allow performing parametric analysis on cost data.

### 4.6. How Significant Is the Burden?

Comparing costs to CHE and GDP was used to show the extent of the burden. Taking the UK as an example; the amount of extra costs that a family of a disabled child bear annually (≈$2600–4200) [33] is comparable to the CHE per capita (61–98%). From the societal perspective, the annual costs of one disability case (ASD) [38] is equal to the country’s GDP per capita. In other words, the society loses one GDP per capita annually for each disabled child. A similar conclusion can be reached if we compare the annual societal costs to the GDP per capita in the Netherlands [50] (108%), Canada [47] (129%), and Sweden [44] (130%). In the Netherlands*,* Hoving et al. estimated that the annual societal costs of one CP child is 11 times CHE per capita [50].

A significant proportion of this burden falls on the families, even in countries with very good public services. In Canada, Genereaux et al. estimated that even with widely available public healthcare and social services, the governmental benefits compensated for less than 6% of OOP expenses that the family of a disabled child face [47].

In developing countries, the burden is even heavier and a significant proportion of it falls on the families. In Mexico, the OOP expenditures incurred by the disabled (DS) child’s family is equal to the county’s CHE per capita [48]. In China, the household expenses were found to be two to eight times the CHE per capita (for mental disability and autism respectively) [52], while according to another study, ASD costs could be up to 17 times CHE per capita (75% of the GDP per capita) [37]. This considerable burden on the households explains why many families fall into poverty and incur catastrophic health expenditures as a result of having a disabled child. This shows how significant the burden of childhood disability but also hints at the potential savings from prevention programs.

### 4.7. Strengths and Limitations

This study is the first to analyse in detail the methods of estimating childhood disability costs differentiating between methods according to perspective, time horizon, type of disability, and country income level.

This review however had some limitations. The heterogeneity of methods did not allow costs to be summarized and compared statistically in a meta-analysis. Time constrains lead to limiting database searches to only four databases. As only one researcher was performing the review, it was not possible to have two independent researchers to select studies according to inclusion criteria.

Search terms used to search the term “disability” included some general synonyms of the term “disability” and terms for some specific medical conditions. However, the terms used for specific medical condition was not a comprehensive list. It only included some of the most common childhood conditions causing disability, while some other conditions (e.g., hearing-impairment and visual-impairment) were not included.

Only five studies from developing countries were included in this review. This was due to the limited number of available studies and their lower quality. Only two studies from developing countries had a low risk of bias according to our critical appraisal. However, a further three studies, who had moderate risk of bias, were included in our review to allow comparison between developed and developing countries.

### 4.8. Recommendations for Policy Makers

Policy makers should look at childhood disability from a wider perspective and consider the effects beyond the health system to appreciate the full picture and avoid underestimation of the economic burden. This is important when making investment decisions and weighing costs and benefits.

Governments should be encouraged to finance programs that are proven effective in preventing avoidable disabilities. Providing facilities and personnel to ensure safer deliveries, especially in rural areas, can significantly decrease the number of CP cases caused by antenatal complications. A facility/program for safe delivery in a developing country can prevent hundreds of traumatic CP cases and save considerable potential lifelong costs of their disability. Some medications to prevent premature delivery are also promising in reducing CP because of prematurity. Amniocentesis for detection of DS and counselling for positive mothers should be provided by public facilities. Early diagnosis, rehabilitation, special education programs, and inclusion in the labor market are effective in improving the outcome of ASD cases. Investing in these interventions with the target of making autistic children more independent and to qualify them to enter the labor market will significantly reduce the lifetime costs of their disability. The same can be concluded for interventions, equipment, and programs to help children with hearing, visual, or physical impairment to overcome their disability and reduce the burden on their family and society. The cost of providing cochlear implants for hearing-impaired children at a young age to make them pursue almost normal academic achievement cannot be compared to the cost of living with lifelong permanent hearing impairment if the operation is delayed. Cochlear implantation is a relatively expensive operation, but the annual loss of one GDP per capita is much more costly to society. This is the argument that should be presented to policy makers to persuade them to take a more societal and long-term viewpoint when making a decision to pay for an immunization program, rehabilitation facility, safe labor initiative or subsidising prosthetics.

Policies in developing countries should aim—beside prevention programs—to reduce the economic burden on the families by providing/subsidising services that the families must pay OOP. Special subsidised insurance schemes and arrangements can be made to allow the children to receive services from private providers if they are not available in public facilities.

Donor organizations should be aware of the magnitude of the economic burden and the potential for preventing it. They should be encouraged to fund programs and services that reduce the OOP expenses by the households to prevent catastrophic expenditure.

Legislation should be passed to encourage employers to allow more disabled persons to enter the labor market.

### 4.9. Need for Further Research

More research is needed on the cost-effectiveness of interventions and prevention programs for childhood disabilities especially in developing countries. The cost of illness studies like those included in this review can be used as inputs in such cost-effectiveness studies.

More cost of disability studies in developing countries are needed. These studies need to include more details on how costs are calculated and become more explicit in their methodology. A societal perspective should be adopted to show the extent of the burden beyond the family and the health system.

Studies to establish the relation between poverty and disability in developing countries are also needed.

## 5. Conclusions

All reviewed studies on the cost of childhood disability—despite their methodological heterogeneity—have reached the conclusion that childhood disability causes a considerable burden on family, health system, and society. Childhood disability could be costing societies up to one GDP per capita annually for each disabled child.

This study could not combine the estimated costs into one measure because the studies were not uniform in the costs they included, sources of costs, and methods of calculation. In addition, perspective, study setting, time horizon, and type of disability are all variables that affect which costs are included and how they are calculated. However, standardizing the way we evaluated these costs in relation to the country’s CHE and GDP allowed us to display the results in a graphical way to allow better understanding of the global picture of the burden of childhood disability. Societies in developed countries pay high costs because of childhood disabilities, and families in developing countries are forced below the poverty line by spending most of their income on their disabled child.

Policy makers should be made aware of the heavy consequences of childhood disability, and search for efficient ways to mitigate these effects. Interventions that have already proven effective in preventing disability should be adopted to prevent the occurrence of disability altogether. For non-preventable disabilities; policy makers must ensure the provision of healthcare, special education, and social services for disabled children and their families to alleviate some of the burden on them and allow them to enter the workforce and be more productive. Providing these services, besides being a moral and ethical obligation on more-abled members of society, is reflected positively in the economy by decreasing the societal costs of disabled children and increasing their household productivity.

Research from developing countries is low in quantity and quality. Researchers in developing countries are required to produce more evidence and be clear on details of methods used and how costs were calculated. Careful analysis of the context and the health system of the study setting is essential to reach the correct decisions regarding the perspective of the study and what costs to include. Economic evaluation of prevention programs and therapeutic interventions is needed to highlight potential savings by preventing childhood disabilities or modifying their effect.

## Figures and Tables

**Table 1:** Results of the literature review on costs of childhood disability.

First Author and Year of Publication	Setting	Perspective	Time Horizon	Medical Condition	Costs in 2019 US$	CHE per Capita 2019 US$ (% of Estimated Costs) *	GDP per Capita 2019 US$ (% of Estimated Costs) **
Solmi [33], 2018	UK, Developed	Household	Weekly	Mentally Disabled	Annual 3625	4267 (85%)	42,310 (9%)
				Severely Disabled	Annual 4200	4267 (98%)	42,310 (10%)
				Physically Disabled	Annual 2600	4267 (61%)	42,310 (6%)
Knapp [38], 2009	UK, Developed	Societal	Lifetime	ASD	Lifetime 2,060,060		
					Annual ^†^ 42,539	4267 (997%)	42,310 (101%)
Ganz [39], 2007	USA, Developed	Societal	Lifetime	ASD	Lifetime 4,345,600		
					Annual 69,530	10,640 (653%)	64,767 (107%)
Wilson [43], 2005	USA, Developed	Societal	Annual	Chronically ill	Annual 36,310	10,640 (341%)	64,767 (56%)
				HIV positive	Annual 13,039	10,640 (123%)	64,767 (20%)
Kruse [40], 2009	Denmark, Developed	Societal	Lifetime	CP	Lifetime 1,344,340 men 1,250,550 women		
					Annual 19,205 men 17,865 women	6000 (320%) (298%)	59,999 (32%) (30%)
Jarbrink [44], 2007	Sweden, Developed	Societal	Annual	ASD	Annual 68,863	6156 (1119%)	53,004 (130%)
Geelhoed [45], 2011	Australia, Developed	Health system	Annual	DS	Annual 3602	5393 (67%)	55,421 (6%)
Roddy [46], 2019	Ireland, Developed	Societal	Annual	ASD	Annual 49,867	5130 (972%)	76,911 (65%)
Wang [42], 2008	China, Developing	Societal	Lifetime	CP	Lifetime 91,046		
					Annual 2365	429 (551%)	10,153 (23%)
Genereaux [47], 2015	Canada, Developed	Societal	Annual	IDD	Annual 60,059	4806 (1250%)	46,419 (129%)
Martınez-Valverde [48], 2019	Mexico, Developing	Household	Annual	DS	Annual 501	498 (101%)	9858 (5%)
Newacheck [49], 2004	USA, Developed	Health system	Annual	Multiple	Annual 3833	10,640 (36%)	64,767 (6%)
Hoving [50], 2007	Netherlands, Developed	Societal	Annual	CP	Annual 57,383	5112 (1123%)	53,016 (108%)
Kageleiry [35], 2016	USA, Developed	Health system	Childhood (0–18 yrs)	DS	0–18 yrs 275,603		
					Annual 15,311	10,640 (144%)	64,767 (24%)
Park [41], 2011	South Korea, Developed	Health system	Lifetime	CP	Lifetime 32,003		
					Annual 457	2203 (21%)	30,028 (1%)
Barrett [34], 2015	UK, Developed	societal	6 months	Autistic Disorder	Annual 35,100	4267 (823%)	42,310 (83%)
				Special Educational Needs	Annual 29,503	4267 (691%)	42,310 (70%)
				ASD	Annual 28,548	4267 (669%)	42,310 (67%)
Peng [51], 2009	USA, Developed	Health system	Annual	PDD	Annual 10,538	10,640 (99%)	64,767 (16%)
Griffiths [36], 2012	Senegal, Developing	Household	Lifetime	Bacterial Meningitis	Lifetime 41,230		
					Annual 1374	58 (2370%)	1510 (91%)
Xiong [52], 2011	China, Developing	Household	Annual	Autism	Annual 3566	429 (831%)	10,153 (35%)
				Physical Disability	Annual 2989	429 (697%)	10,153 (29%)
				Mental Disability	Annual 1164	429 (271%)	10,153 (11%)
Ou [37], 2015	China, Developing	Household	Annual	ASD	Annual 7470	429 (1741%)	10,153 (74%)

* % = (annual costs/current health expenditure (CHE) per capita). ** % = (annual costs/gross domestic product (GDP) per capita). Studies are arranged by strength of evidence according to our critical appraisal tool, the highest quality at the top. ASD = Autism Spectrum Disorders, CP = Cerebral Palsy, DS = Down Syndrome, IDD = Intellectual Developmental Disorder, PDD = Pervasive Developmental Disorder. Care must be taken not to simply compare the costs from different studies as there are considerable methodological differences between them, this table is just a way of summarizing these differences. ^†^ The study by Knapp et al estimated both annual costs (survey based) and modelled lifetime costs (model based). Unlike the rest of lifetime studies in this review, what we are presenting for this study as annual cost is the number reported by Knapp not our calculation from dividing lifetime costs by number of years in the model.

## Data Availability

This is a literature review; data is contained within the article.

## Appendix A. Search Strategy for OVID Medline Database

Ovid MEDLINE(R) and Epub Ahead of Print, In-Process and Other Non-Indexed Citations, Daily and Versions(R) <1946 to 03 July 2019>

1. (cost* adj5 (economic or illness or sickness or disease* or care or direct or indirect)).mp. [mp=title, abstract, original title, name of substance word, subject heading word, floating sub-heading word, keyword heading word, organism supplementary concept word, protocol supplementary concept word, rare disease supplementary concept word, unique identifier, synonyms] (127447)
2. exp “cost of illness”/ or exp healthcare costs/ (82347)
3. 1 or 2 (146362)
4. (disable* or disabilit* handicap* or impairment* paralys* paralyz*).mp. [mp=title, abstract, original title, nameof substance word, subject heading word, floating sub-heading word, keyword heading word, organism supplementary concept word, protocol supplementary concept word, rare disease supplementary concept word, unique identifier, synonyms] (77792)
5. exp Disabled Persons/ (62208)
6. cerebral palsy.mp. [mp=title, abstract, original title, name of substance word, subject heading word, floating sub-heading word, keyword heading word, organism supplementary concept word, protocol supplementary concept word, rare disease supplementary concept word, unique identifier, synonyms] (26008)
7. exp cerebral palsy/ (19602)
8. (down* syndrome or mongolism or trisomy?21).mp. [mp=title, abstract, original title, name of substance word, subject heading word, floating sub-heading word, keyword heading word, organism supplementary concept word, protocol supplementary concept word, rare disease supplementary concept word, unique identifier, synonyms] (29343)
9. exp down syndrome/ (23636)
10. autis*.mp. [mp=title, abstract, original title, name of substance word, subject heading word, floating sub-heading word, keyword heading word, organism supplementary concept word, protocol supplementary concept word, rare disease supplementary concept word, unique identifier, synonyms] (46566)
11. exp Autistic Disorder/ or exp Autism Spectrum Disorder/ (26617)
12. 4 or 5 or 6 or 7 or 8 or 9 or 10 or 11 (187674)
13. (child* or boy* or girl* or adolescent* or infant* or pediatric* or paediatric or (schoolage* or (school adj1 age*)) or (preschool* or (pre adj1 school*))).mp. [mp=title, abstract, original title, name of substance word, subject heading word, floating sub-heading word, keyword heading word, organism supplementary concept word, protocol supplementary concept word, rare disease supplementary concept word, unique identifier, synonyms] (3934927)
14. exp child/ or exp Disabled Children/ or exp Child Health Services/ (1841396)
15. 13 or 14 (3935165)
16. 3 and 12 and 15 (1121)
17. limit 16 to (english language and yr=“1980 -Current”) (1046)

## Appendix B. Search Strategy for OVID Embase Database

Embase Classic+Embase <1947 to 2019 July 03>

1. (cost* adj5 (economic or illness or sickness or disease* or care or direct or indirect)).mp. [mp=title, abstract, heading word, drug trade name, original title, device manufacturer, drug manufacturer, device trade name, keyword, floating subheading word, candidate term word] (284046)
2. exp “cost of illness”/ or exp health care costs/ (291451)
3. 1 or 2 (370332)
4. (disable* or disabilit* handicap* or impairment* paralys* paralyz*).mp. [mp=title, abstract, heading word, drug trade name, original title, device manufacturer, drug manufacturer, device trade name, keyword, floating subheading word, candidate term word] (63633)
5. exp Disabled Persons/ (57465)
6. cerebral palsy.mp. [mp=title, abstract, heading word, drug trade name, original title, device manufacturer, drug manufacturer, device trade name, keyword, floating subheading word, candidate term word] (41772)
7. exp cerebral palsy/ (38329)
8. (down* syndrome or mongolism or trisomy?21).mp. [mp=title, abstract, heading word, drug trade name, original title, device manufacturer, drug manufacturer, device trade name, keyword, floating subheading word, candidate term word] (40125)
9. exp down syndrome/ (36240)
10. autis*.mp. [mp=title, abstract, heading word, drug trade name, original title, device manufacturer, drug manufacturer, device trade name, keyword, floating subheading word, candidate term word] (69358)
11. exp Autistic Disorder/ or exp Autism Spectrum Disorder/ (63878)
12. 4 or 5 or 6 or 7 or 8 or 9 or 10 or 11 (230190)
13. (child* or boy* or girl* or adolescent* or infant* or pediatric* or paediatric or (schoolage* or (school adj1 age*)) or (preschool* or (pre adj1 school*))).mp. [mp=title, abstract, heading word, drug trade name, original title, device manufacturer, drug manufacturer, device trade name, keyword, floating subheading word, candidate term word] (4146231)
14. exp child/ or exp Disabled Children/ or exp Child Health Services/ (2909761)
15. 13 or 14 (4427076)
16. 3 and 12 and 15 (1415)
17. limit 16 to (english language and yr=“1980-Current”) (1326)

## Appendix C. Search Strategy for OVID Econlit Database

Econlit <1886 to June 27, 2019>

1. (disable* or disabilit* handicap* or impairment* paralys* paralyz*).mp. [mp=heading words, abstract, title, country as subject] (1187)
2. cerebral palsy.mp. [mp=heading words, abstract, title, country as subject] (9)
3. (down* syndrome or mongolism or trisomy?21).mp. [mp=heading words, abstract, title, country as subject] (10)
4. autis*.mp. [mp=heading words, abstract, title, country as subject] (70)
5. (child* or boy* or girl* or adolescent* or infant* or pediatric* or paediatric or (schoolage* or (school adj1 age*)) or (preschool* or (pre adj1 school*))).mp. [mp=heading words, abstract, title, country as subject] (39334)
6. (cost* or burden*).mp. [mp=heading words, abstract, title, country as subject] (191228)
7. 1 or 2 or 3 or 4 (1268)
8. 5 and 6 and 7 (44)
9. limit 8 to (yr=“1980-Current” and english) (42)

## Appendix D. Search Strategy for OVID Global Health Database

Global Health <1910 to 2019 Week 26>

1. (cost* adj5 (economic or illness or sickness or disease* or care or direct or indirect)).mp. [mp=abstract, title, original title, broad terms, heading words, identifiers, cabicodes] (24319)
2. exp “cost of illness”/ or exp health care costs/ (11630)
3. estimated costs/ or health care costs/ or costs/ or social costs/ or unit costs/ or specific costs/ or total costs/ (26116)
4. exp “cost analysis”/ or exp costs/ or exp “cost effectiveness analysis”/ (30831)
5. 1 or 2 or 3 or 4 (38096)
6. (disable* or disabilit* handicap* or impairment* paralys* paralyz*).mp. [mp=abstract, title, original title, broad terms, heading words, identifiers, cabicodes] (6019)
7. exp Disabled Persons/ (4887)
8. cerebral palsy.mp. [mp=abstract, title, original title, broad terms, heading words, identifiers, cabicodes] (1407)
9. exp cerebral palsy/ (1061)
10. (down* syndrome or mongolism or trisomy?21).mp. [mp=abstract, title, original title, broad terms, heading words, identifiers, cabicodes] (1981)
11. autis*.mp. [mp=abstract, title, original title, broad terms, heading words, identifiers, cabicodes] (2467)
12. disabilities.sh. (7353)
13. exp disabilities/ or exp people with disabilities/ (11117)
14. cerebral palsy.sh. (1061)
15. exp autism/ (1831)
16. Down’s syndrome.sh. (1623)
17. 6 or 7 or 8 or 9 or 10 or 11 or 12 or 13 or 14 or 15 or 16 (18089)
18. (child* or boy* or girl* or adolescent* or infant* or pediatric* or paediatric or (schoolage* or (school adj1 age*)) or (preschool* or (pre adj1 school*))).mp. [mp=abstract, title, original title, broad terms, heading words, identifiers, cabicodes] (530913)
19. exp children/ (339666)
20. 18 or 19 (530913)
21. 5 and 17 and 20 (108)
22. limit 21 to (english language and yr=“1980-Current”) (89)

## Appendix E

**Table A1:** Checklist Used for Critical Appraisal.

	Yes	No	N/A
Was a well-defined question posed in answerable form?			
Did the study examine costs of the service(s) or programme(s)?			
2. Did the study involve a comparison group?			
3. Was a viewpoint for the analysis stated and was the study placed in any particular decision-making context?			
4. Were the patient population and any relevant subgroups adequately defined?			
Were all the important and relevant costs identified?			
5. Was the range wide enough for the research question at hand?			
6. Did it cover all relevant viewpoints? (Possible viewpoints include the community or social viewpoint, and those of patients and third-party payers. Other viewpoints may also be relevant depending upon the particular analysis.)			
7. Were the capital costs, as well as operating costs, included?			
Were costs measured accurately in appropriate physical units (e.g., hours of nursing time, number of physician visits, lost work-days)?			
8. Were any of the identified items omitted from measurement? If so, does this mean that they carried no weight in the subsequent analysis?			
9. Were there any special circumstances (e.g., joint use of resources) that made measurement difficult? Were these circumstances handled appropriately?			
Were the costs valued credibly?			
10. Were the sources of all values clearly identified? (Possible sources include market values, patient or client preferences and views, policy-makers’ views and health professionals’ judgements)?			
11. Were market values employed for changes involving resources gained or depleted?			
12. Where market values were absent (e.g., volunteer labour), or market values did not reflect actual values (such as clinic space donated at a reduced rate), were adjustments made to approximate market values?			
Were costs adjusted for differential timing?			
13. Were costs that occur in the future ‘discounted’ to their present values?			
14. Was there any justification given for the discount rate used?			
Was uncertainty in the estimates of costs adequately characterized?			
15. If patient-level data on costs available, were appropriate statistical analyses performed?			
16. Were the conclusions of the study sensitive to the uncertainty in the results, as quantified by the statistical analysis?			
17. Was heterogeneity of the patient population recognized, for example by presenting study results for relevant subgroups?			
Did the presentation and discussion of study results include all issues of concern to users?			
18. Were the results compared with those of others who have investigated the same question? If so, were allowances made for potential differences in study methodology?			
19. Did the study discuss the generalisability of the results to other settings and patient/client groups?			
20. Were the implications of uncertainty for decision-making, including the need for future research, explored?			

## Appendix F

**Table A2:** Data Extraction.

Author and Setting	Research Question	Perspective and Time Horizon	Medical Condition	Method of Disability Definition	Method(s) of Calculating Cost	Summary of the Main Findings/2019 Dollars	Comments
Francesca Solmi [33], 2018, UK, Developed	to quantify the cost of mental and physical disability in childhood and adolescence to families in the UK	household, weekly	multiple	Disability Discrimination Act definition	Compensating Variation	Annual additional costs to households with a mentally disabled child ≈$3600, for severely disabled child ≈$4200 and for physically disabled children ≈$2600	New approach for estimating burden of disability
Martin Knapp [38], 2009, UK, Developed	to estimate the societal costs of Autism Spectrum Disorders (ASDs) in the UK by combining national data	societal, lifetime	ASD	Confirmed diagnosis of Autism or Pervasive Developmental Disorder (PDD) from a variety of tests and instruments in the original studies	modular approach from several sources to obtain nationally representative data cost per individual*:* service use data from recent studies lost productivity: estimated from national estimates of average weekly wage for full-time employees at April 2005 (£431)	Average annual total cost of supporting a child with ASDs ≈$42,500 Lifetime costs of a child with ASD and intellectual disability ≈$2 million	
Michael L. Ganz [39], 2007, USA, Developed	To estimate lifetime incremental societal costs of autism in the United States	societal, lifetime	ASD	multiple sources	***(A prevalence-based cohort)*** cross-sectional cost data from different age groups were used to create prevalence-based cost estimates that approximate incidence-based estimates **Direct medical and non-medical costs:** obtained from the literature. Indirect costs: computed using ***human capital approach***. Costs were projected across the life span, and discounted, then incremental age-specific costs were computed	The lifetime per capita incremental societal cost of autism is ≈$4.3 million	
Leslie S. Wilson [43], 2005, USA, Developed	compare types, amounts, and costs of care provided toHuman Immune deficiency Virus HIV-positive children and chronically ill children, using healthy children to control for basic care needs	societal, annual	multiple	Not clear	***Human capital approachMarket replacement method*** to calculate the value of lost opportunity	Annual costs for homecare for chronically ill child ≈$36,000 and for HIV positive child ≈$13,000 Informal caregiving represents a substantial economic value to society	Provides good details on estimating opportunity costs
Marie Kruse [40], 2009, Denmark, Developed	to quantify the average societal costs of Cerebral Palsy (CP) per individual over a lifetime in Denmark	societal, lifetime	CP	CP cases registered in the National registry	Quantified health care, productivity, and social care costs by means of ***register-based data, in a cross-sectional perspective***. Data for CP cases were linked to the information of national registers by the unique personal identifier, allowing identification of costs attributable to CP. **Productivity loss** estimated by ***human capital approach*** **Social care costs:** estimated from published information on average costs per user, and validated by a group of experts.	The lifetime costs of CP were fo males ≈$1.35 million and females ≈$1.25 millionhigh social care costs and productivity costs associated with CP Healthcare costs are only 7% of total lifetime societal costs	Good details on methods of calculating each type of cost. The unique personal identifier used in all Danish registers allows comprehensive register linkages and vast analysis opportunities.
Krister Jarbrink [44], 2007, Sweden, Developed	to describe and evaluate the societal costs of ASD for children living in a Swedish municipality	societal, annual	ASD	Confirmed diagnosis of ASD	Data collected from service use and combined with unit **costsproductivity loss**, by ***human capital approach*** (lost hour of work was valued using the average gross wage) The unit cost for valuation of losses in time for unpaid work and leisure time was set to 35 percent of the average gross wage	Additional annual societal cost due to ASD ≈$69,000 Main cost drivers are the community support services and schooling	very good methodology for estimating societal costs mall sample size
Elizabeth A. Geelhoed [45], 2011, Australia, Developed	To assess the direct annual health care costs for children and adolescents with Down syndrome (DS) in Western Australia	health system, annual	DS	children/young adults registered in Disability Services Commission with DS	**Resource items:** from survey reported by the family, computed to units per year, and costed according to the unit price **Medical costs:** from the Medical Benefit **ScheduleHome and community care services:** from the Disability Services Commission **Hospital admission costs:** mean bed day cost**Medications:** Schedule of Pharmaceutical Benefits **Complementary medicines, non-durable health-related products, and therapeutic devices:** based on expenditure provided by the parents	The total mean annual health care costs across all age groups was ≈$3600, with a median of ≈$1500Overall, costs decrease with age with most costs in the first two years of life	Good details of how the cost of each item was calculated Costs presented in both ***mean***to represent the total cost burden, and ***median*** to represent expected cost for an individual
Aine Roddy [46], 2019, Ireland, Developed	Estimate the societal cost of childhood ASDs and explain the variation in costs between state and family Out-Of-Pocket (OOP) expenditure	societal, annual	ASD	from autism organizations database and social media!	Data collected through an online survey to parents***A bottom-up prevalence based cost-of-illness methodology adopting a societal perspective*** Resources consumed: multiplied by unit costs then summed to produce individual aggregate costs **Parental OOP expenditure:** only attributable to ASD Informal care costs: only for parents who gave up employment because of their child’s condition using a ***human capital approach***based on the industrial wage per hour	Annual average family OOP costs for ASD case ≈$33,500 Annual average state expenditure per ASD child ≈$16,500	good details on costing methods, systematic NO control group /low response rate /convenience sample, may not representative
Bin Wang [42], 2008, China, Developing	Measure the economic burden of CP in China and its societal impact	societal, lifetime	CP	Confirmed CP diagnostic documentation from a secondary or tertiary hospital	Estimates were obtained from ***interviewing caregivers*** of individuals with CP ***Human capital approach*** for indirect cost *Incidence approach* rather than prevalenceservice demand rather than need	Average lifetime economic burden of a new CP case born in China in 2003 ≈$91,000 Indirect (productivity) costs are responsible for 93% of total economic loss, and direct healthcare and developmental costs make up 3% each	sample bias is likely as they only recruited 319 patients (all admitted in hospitals) because there is no registry
Dallas Genereaux [47], 2015, Canada, Developed	estimate OOP costs to parents, and the non-health system costs to society, of raising a child with intellectual developmental disorder (IDD)	societal, annual	multiple	Confirmed diagnosis of IDD of unknown cause	**Directly reported costs:** taken at face value **Opportunity costs:** by ***human capital approach*** (market-replacement method based on job classification) **Non-hospital therapies:** cost per encounter method using labor-market wages for the position **Non-parent volunteered care:** labor market average rate **Income loss by parents:** hourly wage or yearly income based on their current or most recent job	Median annual **parental costs** of caring for an IDD child ≈$37,000 Median annual non-health system societal costs ≈$23,000 **Main parental cost drivers** = income loss and care giving time **Main societal cost driver** = special educationParental and societal costs increase with IDD severity Parental costs are not adequately compensated by government	Very nuanced description of how each cost was estimated Small sample size, no comparison group
Silvia Martınez-Valverde [48], 2019, Mexico, Developing	Investigate the burden of OOP household expenditures and time spent on care by families responsible for children with DS	household, annual	DS	Confirmed diagnosis of DS in the outpatient clinic of the genetics department	Cross-sectional analysis based on survey of families of DS childrenTotal OOP expenditures = medical care and transportation for one year Available household expenditures = total household expenditures—the cost of food and housing Catastrophic expenditures = if total OOP on medical care and transportation >30% of available household expenditures	67% of the households with children with DS were within the lower four deciles (I–IV) of expenses, indicating a limited ability to pay for medical services. Yearly OOP expenditures for a child with DS represented 27% of the available household expenditure, which is equivalent to ≈$500 33% of families with DS children had catastrophic expenses 46% of the families had to borrow money to pay for medical expenses	Highlight differences in the health system between developed vs developing countries where the family pays a lot of OOP
Paul W. Newacheck [49], 2004, USA, Developed	To examine health care utilization and expenditure patterns for children with disabilities	health system, annual	multiple	Bio-psycho-social approach	not clear if self-reported or from insurance claims!	children with disabilities had much higher health care expenditures ≈$3800 than non-disabled ≈$676 and higher OOP expenditures Low-income families are especially vulnerable to burdensome OOP	
M A Hoving [50], 2007, Netherlands, Developed	Estimate and categorize the expenditures for children with intractable spastic CP	societal, annual	CP	Diagnosis with intractable spastic CP	**Bottom-up, disease specific, prevalence-based cost-of-illness study**	From a societal perspective, mean annual costs were ≈$57,000 more than 11 times the CHE per capita 95% of costs are outside the medical care sector The societal costs are 71% paid by the health insurers, 24% by the government, 5% by the families	
Andrew Kageleiry [35], 2016, USA, Developed	To estimate incremental medical costs for DS children, up to the age of 18 years in the USA	health system, 0-18 years	DS	International Classification of Diseases, Ninth revision, Clinical Modification (ICD-9-CM) diagnosis of DS	Claim database was used to conduct ***retrospective cohort study*** **OOP expenses:** from claims data (co-pay and co-insurance) The observation period for each individual was split into clinically relevant age categories	Average total incremental medical costs for a privately insured DS child from birth till 18 years of age ≈$275,000 of which ≈$20,000 are OOP expenses incurred by the family	Only direct OOP not including premium and not including education, transportation. very narrow scope
Moon Seok Park [41], 2011, South Korea, Developed	lifetime healthcare costs attributable to cerebral palsy in S. Korea	health system, lifetime	CP	CP diagnosis by Korean Standard Disease Classification Codes	**Medical cost data** attributable to CP was retrieved from claims data of the national health insurance according to the disease classification codes CP	The attributable lifetime medical cost of CP in South Korea was ≈$32,000, which is 1.8 times the basic lifetime medical cost of the general population	Lifetime cost calculated only from health system perspective but no direct non-medical cost / no OOP / no indirect costs or lost productivity Narrow scope for lifetime
Barbara Barrett [34], 2015, UK, Developed	Utilization and costs for: adolescents with autistic disorder, adolescents with other ASDs, adolescents with other special educational needs and typically developing adolescents	societal, 6 months	ASD	Clinically diagnosed ASD according to ICD-10	**Resource use data:** self-reported via interview of parents **Total costs:** multiply each resource used by unit costs of public services in UK	Costs per individual were highest in the autistic disorder group (≈$35,100), followed by the special educational needs group (≈$29,500), the broader autism spectrum disorder group (≈$28,500) and the typically developing group (≈$9500)	STRENGTH: There is comparison group/highlights significant cost of educational services in ASD WEAKNESS: no indirect family cost, no OOP costs calculated
Chun-Zi Peng [51], 2009, USA, Developed	To estimate healthcare utilization rates and cost of care for children with pervasive developmental disorders (PDD)	health system, annual	multiple	Children with PPD diagnosis by ICD-9	Costs were calculated by summing the claim charges.	Average annual medical cost for children with PDD ≈$10,500, eight times that of normal children	Claims analysis with only direct healthcare data not including OOP/education/other non-medical related expenditures/no societal cost
Ulla K. Griffiths [36], 2012, Senegal, Developing	estimate the costs of meningitis sequelae in children in Senegal from the perspective of households	household, lifetime	Bacterial meningitis	“sequela” was defined according to the 2006 Global Burden of Disease project	***Self-reported*** by caregivers even ***for lost******productivity*** i.e., income foregone (more appropriate to low-income settings than the human capital or the friction cost approach)	Lifetime cost of meningitis sequelae (≈$41,000) is approximately 26 times higher than the mean cost of treating an acute meningitis episode	The first study that has assessed the costs of meningitis sequelae from the perspective of households in a low-income setting.
Nina Xiong [52], 2011, China, Developing	To estimate family costs of raising autistic, physically disabled, or mentally disabled children	household, annual	multiple	Not clear	**Formula:** the raising burden of children with disability = [(the income per person per year of family of normal children—that of family of disabled children) + (the cost per person per year of disabled children—that of normal children)—(the economic assistance per family per year of disabled children—assistance of normal children)]	Annual average raising burden of children with autism ≈$3600, physical disability ≈$3000, mental disability ≈$1200	
Jian-Jun Ou [37], 2015, China, Developing	evaluate the employment and financial burdens of families with ASD-diagnosed pre-schoolers	household, annual	ASD	diagnosed with ASD in a local hospital	***A questionnaire with open questions*****:** (How much total income do you expect that all family members would have earned in the past year if your child did not have the disease?) (What were the total educational expenses of the ASD or OD child in your household over the past year, including general/special school education, extracurricular practice, related books/materials, and so on?)	The average loss of annual income associated with having a child with ASD was ≈$7500, compared with ≈$3500 for families of OD children	Opportunity cost is measured through parents’ reporting rather than the typical human capital method
